# Ultraviolet-C Irradiation: A Novel Pasteurization Method for Donor Human Milk

**DOI:** 10.1371/journal.pone.0068120

**Published:** 2013-06-26

**Authors:** Lukas Christen, Ching Tat Lai, Ben Hartmann, Peter E. Hartmann, Donna T. Geddes

**Affiliations:** 1 School of Chemistry and Biochemistry, Faculty of Science, The University of Western Australia, Crawley, Western Australia, Australia; 2 Carag AG, Baar, Switzerland; 3 Perron Rotary Express Milk Bank, King Edward Memorial Hospital, Subiaco, Western Australia, Australia; 4 Centre for Neonatal Research and Education, The University of Western Australia, Crawley, Western Australia, Australia; Louisiana State University and A & M College, United States of America

## Abstract

**Background:**

Holder pasteurization (milk held at 62.5°C for 30 minutes) is the standard treatment method for donor human milk. Although this method of pasteurization is able to inactivate most bacteria, it also inactivates important bioactive components. Therefore, the objective of this study was to investigate ultraviolet irradiation as an alternative treatment method for donor human milk.

**Methods:**

Human milk samples were inoculated with five species of bacteria and then UV-C irradiated. Untreated and treated samples were analysed for bacterial content, bile salt stimulated lipase (BSSL) activity, alkaline phosphatase (ALP) activity, and fatty acid profile.

**Results:**

All five species of bacteria reacted similarly to UV-C irradiation, with higher dosages being required with increasing concentrations of total solids in the human milk sample. The decimal reduction dosage was 289±17 and 945±164 J/l for total solids of 107 and 146 g/l, respectively. No significant changes in the fatty acid profile, BSSL activity or ALP activity were observed up to the dosage required for a 5-log_10_ reduction of the five species of bacteria.

**Conclusion:**

UV-C irradiation is capable of reducing vegetative bacteria in human milk to the requirements of milk bank guidelines with no loss of BSSL and ALP activity and no change of FA.

## Introduction

Human milk contains nutritional and bioactive components that cannot be provided to the infant by formula milk. Therefore, donor human milk is the best alternative if mothers own milk is not available for the preterm infant [1], [2]. To minimize the risk of transmission of diseases from a donor mother to a preterm infant, the donor human milk is pasteurized. Pasteurisation is defined as a process where food, usually a liquid, is treated to make the product safe for consumption and to increase its shelf-life. Heat treatments are the most common commercial pasteurisation methods but alternative treatments including ultrasound and irradiation are emerging that have the potential to improve the quality of pasteurised donor human milk.

The current pasteurization method used in most human milk banks is a low temperature long time heat treatment (Holder pasteurization). Human milk is heated in a water bath and held for 30 minutes at 62.5°C [3]–[5]. This treatment is capable of a 5-log_10_ reduction of bacteria including *Escherichia coli, Staphylococcus epidermidis, Enterobacter cloacae, Bacillus cereus* and *Staphylococcus aureus* in human milk [6]. However, this process results in the alteration and the loss of activity of important bioactive components. The retention of immunological proteins is significantly reduced and the activity of bile salt stimulated lipase (BSSL) is completely eliminated [6]–[8]. BSSL is a very heat labile enzyme and inactivation starts at 45°C depending on exposure time [9]. BSSL breaks down triacylglycerol into monoacylglycerides and free fatty acids and therefore aids in digestion of fat by the infant [10]. Studies have shown that preterm infants fed pasteurized human milk have a lower fat absorption and growth rate compared to preterm infants fed raw milk [11], [12]. Another enzyme completely lost with Holder pasteurization is alkaline phosphatase (ALP). Due to the fact that alkaline phosphatase is inactivated slightly above the pasteurization conditions required to destroy the targeted micro-organisms in milk it is used in the dairy industry as a biomarker to detect inadequate pasteurization of the milk [13], [14]. Despite its wide use as a biomarker its function in human milk and benefit for newborn infants is not known.

In contrast to total fat concentration, the fatty acid (FA) composition is affected by the mothers diet [15], [16]. Long chain fatty acids (LCFA, 16- to 24-carbon chain) make up 85% of the total fatty acids of human milk and include the omega-3 fatty acid docosahexaenoic acid (DHA) and the omega-6 fatty acid arachidonic acid (AA). DHA and AA are particularly important for infants because of their role in the neural and visual function [17].

Unlike human milk proteins, milk fatty acids, including DHA and AA are not affected by the Holder pasteurization process [8]. Therefore, any alternate non-thermal pasteurization method that is designed to retain other bioactive components in human milk should not result in the loss of important fatty acids.

Ultraviolet (UV) irradiation is classified as a non-thermal disinfection method [18]. UV is electromagnetic radiation and subdivided by wavelength into UV-A (320–400 nm), UV-B (280–320 nm), UV-C (200–280 nm) and Vacuum-UV (100–200 nm). UV-C has the highest germicidal effect, specifically between 250 and 270 nm, and is capable of destroying bacteria, viruses, protozoa, yeasts, moulds and algae [19], [20]. At this germicidal wavelength the DNA bases, mainly pyrimidine and purine, absorb the UV-C energy promoting chemical reactions generating photoproducts. Common photoproducts are pyrimidine dimers, other pyrimidine adducts, pyrimidine hydrates, and may involve cross-links with proteins and on rare occasions breakages of DNA strands [21]. Due to its limited penetrative ability, UV-C irradiation is commonly used in surface sterilization of fruits and vegetables and the treatment of drinking water [22]. The penetration depth of UV-C depends on the solubility, density and turbidity of a liquid [22]–[24]. Milk is difficult to treat with UV-C due to its high absorption coefficient of 300 cm^−1^ at a wavelength of 254 nm compared to the absorption coefficients of drinking water and beer with 0.1 and 20 cm^−1^, respectively [21]. Previous studies showed that UV-C irradiation can be used to successfully reduce the microbial load of opaque liquids such as bovine milk and various fruit juices without affecting sensory quality, however no measurement was made on the bioactivity of milk [25]–[27]. A turbulent flow around a UV-C source was created and this therefore exposed the micro-organisms to photons at the interface between the opaque liquid and the photon source.

The aim of this study was to investigate if an applied vortical flow of human milk around a UV-C source can be used to overcome the limited penetration depth of the rays. Specifically, if a 5-log_10_ bacterial reduction, as required by human milk bank guidelines, can be achieved with a higher retention of BSSL and ALP activity than with Holder pasteurization. In addition, we wished to determine if UV-C treatment affects the fatty acid concentrations in human milk.

## Methods and Materials

### Sample collection and ethics statement

Human milk was donated by ten lactating women (n = 10 samples). Collection of the human milk samples was approved by the Human Research Ethics Committee of The University of Western Australia (RA/4/1/2369). All donors gave written consent for their donations to be used in research and all samples were de-identified. Milk was stored in a −20°C freezer prior to the experiment.

### Sample preparation

Total solids concentrations of human milk samples were adjusted to increase the concentration range. Macronutrient (fat, protein, lactose and total solids) analysis of all samples (n = 10) was determined using a mid-infrared analyser (HMA – Human Milk Analyser, Miris AB, Uppsala, Sweden) [28]. Then, the samples were centrifuged (Allegra X-12R, Beckman Coulter Inc., Brea, CA, USA) and total solids contents adjusted by removing or adding cream from the same sample [29]. The final total solids content of the samples ranged from 107 to 146 g/l (Table 1).

**Table 1:** Total solids, total protein, lactose and fat content of the adjusted human milk samples.

Sample No.	1	2	3	4	5	6	7	8	9	10
Total solids [g/l]	107.0	110.5	120.0	121.0	125.0	126.0	131.5	133.0	139.5	146.0
Total protein [g/l]	7.0	17.5	16.0	20.5	10.0	9.0	15.0	14.0	14.5	10.0
Lactose [g/l]	68.0	72.5	72.5	87.5	71.5	69.5	65.0	65.5	68.5	52.0
Fat [g/l]	26.0	18.0	30.0	10.0	39.0	42.5	50.0	52.0	55.0	81.0

*Staphylococcus epidermidis* (ATCC 12228), *Enterobacter cloacae* (ATCC 27508), *Bacillus cereus* (ATCC 10702) (American Type Culture Collection Inc, Manassas, VA, USA) and *Escherichia coli* K12 (ATCC1498, Southern Biological, Nunawading, Vic, Australia) were cultured in nutrient broth (Nutrient Broth No. 2, Oxoid Australia Pty Ltd, Adelaide, SA, Australia) over night at 33°C. *Staphylococcus aureus* (ATCC 6538) (American Type Culture Collection Inc, Manassas, VA, USA) was cultured in tryptic soy broth (BBL Trypticase Soy Broth, Becton Dickinson & Co, Franklin Lakes, NJ, USA) over night at 33°C. Bacteria were then enumerated using the optical density method with previously constructed standard curves [30]. Cultures were diluted and inoculated into the total solids adjusted human milk samples to a maximum accepted concentration in milk banks of 10^5^ CFU/ml (colony forming units per millilitre) [5].

### Ultraviolet treatment

**Figure 1:**
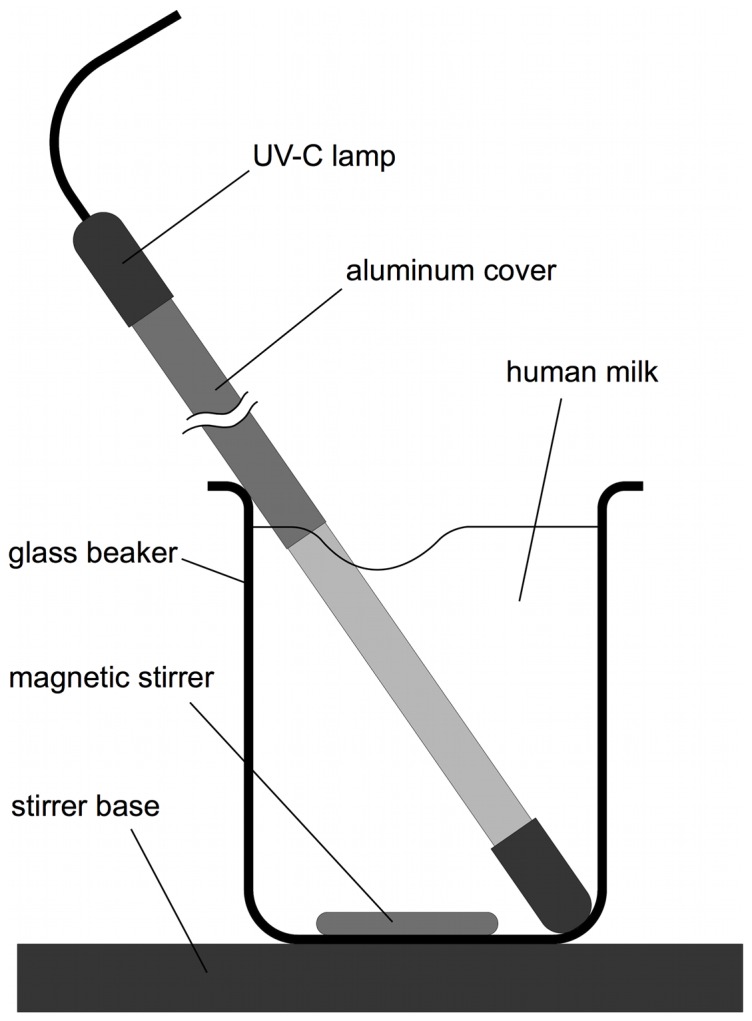
Experimental set-up showing the placement of the UV-C lamp in the human milk sample.

Each human milk sample (380 ml; n = 10) was transferred into a 400 ml PYREX glass beaker No. 1003. A partially covered (57 mm open out of 211 mm arc length) germicidal UV-C lamp (95% of UV-output at 253.7 nm) (GPH287T5L, Infralight Pty Ltd, Helensburgh, NSW, Australia) was placed diagonally into the glass beaker (Figure 1). The UV-C output of the 57 mm open lamp was 1.1 W. During UV-C irradiation, the milk was stirred with a 8×40 mm magnetic stirrer bar at 500 rpm (C-MAG HS 7, IKA, Staufen, Germany) to create a low velocity, laminar flow vortex. The human milk was exposed to UV-C and 1 ml samples were taken at different time points and aliquoted for the different analyses. Dosage (J/l) was calculated as the product of treatment time (s) and UV-C power (W) divided by the treated volume (l). Aliquots for enzymatic and fatty acid analyses were kept frozen at −80°C until the assay was performed. Bacterial analyses were immediately performed and the reduction was measured as decimal reduction dosage, which is the dosage required to reduce the organism by 1-log_10_ (reduction to 10% of the original colony count). For a 5-log_10_ reduction, 5 times the decimal reduction dosage is required and means that the bacterial count is reduced by 99.999%.

### Bacteria analysis

Untreated and UV-C irradiated samples previously inoculated with *E. coli, S. epidermidis, E. cloacae and B. cereus* were plated in duplicates onto nutrient agar (Nutrient Broth No. 2, Oxoid Australia Pty Ltd, Adelaide, SA, Australia/Plain agar, Southern Biological, Nunawading, Vic, Australia) and incubated at 33°C for 18 hours. Untreated and UV-C irradiated samples previously inoculated with *S. aureus* were plated in duplicates onto tryptic soy agar (Difco Tryptic Soy Agar, Becton Dickinson & Co, Franklin Lakes, NJ, USA) and incubated at 33°C for 18 hours. The numbers of colonies were then enumerated to determine the reduction in bacterial number during UV-C irradiation based on colony forming units per millilitre (CFU/ml). Detection limit was at 5 CFU/ml.

### Bile salt stimulated lipase assay

BSSL activity in human milk was determined using a lipase assay kit (QuantiChrom Lipase Assay Kit, BioAssay Systems, Hayward, CA, USA). Frozen (−80°C) human milk samples (n = 10) were thawed and brought to 37°C, diluted with DDI (1/100, v/v), and assayed in duplicate according to kit instructions. The absorbance was determined with a spectrophotometer (PowerWave, Biotek, Winooski, VT, USA) and transformed into BSSL activity per millilitre (U/ml) based on the formula in the kit instructions. Detection range for this kit is between 40 and 1600 U/ml lipase activity in the 96-well plate assay. Unit definition: one unit of enzyme catalyzes the cleavage of 1.0 µM of substrate per minute under the assay conditions (pH 8.5). The coefficient of variation (CV) within groups was <7%.

### Alkaline Phosphatase assay

ALP causes hydrolysis of p-nitrophenyl phosphate and forms a yellow complex whose intensity, measured at 405 nm, is directly proportional to the concentration of ALP in the sample. ALP activity was analysed using the enzymatic assay based on Walter and Schütt [31]. Frozen (−80°C) human milk samples where thawed and brought to 37°C, diluted with DDI (1/4, v/v), 5 µl samples in duplicate were added to 135 µl of substrate (1 M diethanolamine, 0.5 mM MgCl_2_, pH 10.3) into a 96-well plate. Then, 10 µl of the colour reagent (10 mM p-nitrophenyl phosphate) was added into the wells and the plate was incubated at 37°C for 30 minutes. The absorbance was determined with a spectrophotometer at 405 nm (PowerWave, Biotek, Winooski, VT, USA) and transformed into alkaline phosphatase activity per millilitre (U/ml) using a standard curve. Unit definition: one unit of enzyme hydrolyses 1.0 mM of p-nitrophenyl phosphate per minute under the assay conditions. The recovery assay was 94±9% and the intra-assay variation was <6%.

### Fatty acid analysis

FA were analysed using an extraction and trans-esterification method based on Mitoulas et al. [32]. FA were extracted with chloroform/methanol (2/1, v/v) using butylated hydroxyanisole as an antioxidant. FA methyl esters from milk lipid extracts were prepared by acid transmethylation using 1% H_2_SO_4_ in methanol. The fatty acid methyl esters were then separated and quantified by gas chromatography (GC-2010, Shimadzu Co., Kyoto, Japan) using a 50 m capillary column with 0.32 mm inner diameter coated with 0.25 µm BPX-70 (SGE Pty Ltd., Ringwood, VIC, Australia). Each sample (3 µl) was injected onto the column using an automatic injector (AOC-20i, Shimadzu Co., Kyoto, Japan) at a split ratio of 20∶1. The injector temperature was set at 250°C and the detector (flame ionisation) temperature at 300°C. The initial oven temperature was 130°C. The temperature was initially programmed to rise to 155°C within 5 min, then slowly to 165°C within 1 min, then to 230°C within 5 min and finally to 250°C within 10 min where it was held for 5 min. Helium was used as the carrier gas at a velocity of 4 ml/min. Fatty acids were identified based on retention time against authentic lipid standards (Sigma-Aldrich Co., St Louis, MO, USA). Individual FA peaks were quantified as the total area under FA peaks. The coefficient of variation of the area under the FA peaks over 5 separate extractions was <4.2%.

### Statistical analysis

Data analysis was performed using R 2.15.1 for Mac OS X (The R Core Team) [33]. Packages nlme [34] and multcomp [35] were used for linear mixed effects models and multiple comparisons of means, respectively. Graphical representation for the fatty acid concentration was created with the package lattice [36] and the example for bacterial reduction, relationship between decimal reduction dosage and total solids concentraion and alkaline phosphatase retention with LibreOffice 3.5 for Mac OS X (The Document Foundation, Berlin, Germany). *P*<0.05 was considered to indicate significant difference in all analyses. Results are presented as mean ± standard deviation (SD) values unless otherwise stated.

Linear mixed effects models were used to determine if i) reduction of the 5 different bacterial species, ii) BSSL, iii) ALP and iv) FA was different for different UV-C dosages. Total solids was considered as the random effect. Linear mixed effects models were also used to determine if the decimal reduction dosage was different for different total solids concentrations. Individual human milk sample was considered as the random effect. Post-hoc test (Tukey's HSD) was used to identify which levels of the factor was significantly differed.

## Results

### Bacteria analysis

**Figure 2:**
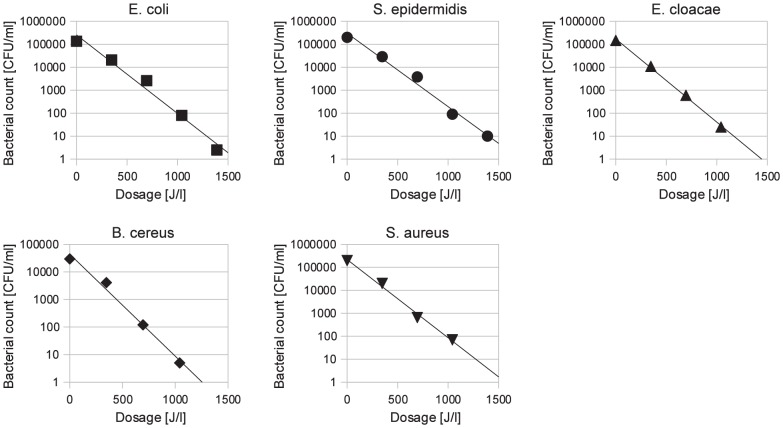
Example of UV-C induced bacterial reduction (total solids concentration of 107 g/l).

Exponential reductions of *E. coli, S. epidermidis, E. cloacae, B. cereus* and *S. aureus* were observed after irradiation with UV-C light (Figure 2). The exponential reduction had a high correlation with the applied dosage (p<0.001). The decimal reduction dosages were not significantly different (p = 0.51) between the five different species of bacteria with mean values of 594±265, 569±209, 565±178, 640±189 and 635±268 J/l, respectively.

**Figure 3:**
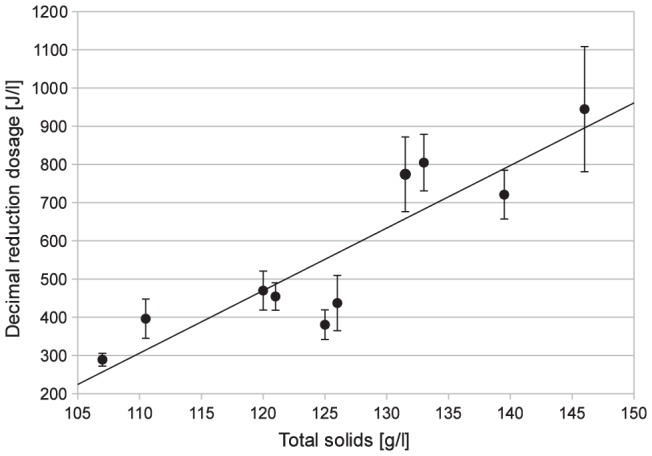
Relationship between bacterial reduction (decimal reduction dosage) and total solids concentration of human milk samples.

There was a strong positive relationship (p<0.001) between the decimal reduction dosage and the total solids concentration in the human milk (Figure 3). Human milk with a total solids concentration of 107 g/l needed a decimal reduction dosage of 289±17 J/l. The decimal reduction dosage needed for a total solids concentration of 146 g/l was over three times higher with 945±164 J/l. For each additional gram per litre of total solids, an additional 16.4 J/l in the decimal reduction dosage was required.

To achieve a 5-log_10_ reduction in 0.38 l of human milk with a total solids concentration of 105, 125 and 145 g/l a treatment time is needed of 8.3, 14.8 and 26.5 min, respectively under these experimental conditions.

### Bile salt stimulated lipase activity

Untreated human milk samples had a mean BSSL activity of 116.5±36.6 U/ml. After treatment with UV-C the BSSL activity did not change significantly (p = 0.24) (Table 2).

**Table 2:** BSSL and ALP activity of untreated and UV-C irradiated samples (mean±SD, n = 10).

UV-C dosage	BSSL activity [U/ml]	ALP activity [U/ml]
untreated	116.5±36.6	0.200±0.050
2084 J/l	112.7±33.3	0.199±0.056
3474 J/l	113.6±33.7	0.199±0.047
4863 J/l	115.3±34.4	0.204±0.057

### Alkaline Phosphatase activity

Untreated human milk samples had a mean ALP activity of 0.200±0.050 U/ml. After treatment with UV-C the ALP activity did not change significantly (p = 0.75) (Table 2).

In an overexposure experiment were the human milk was treated significantly longer than needed to reduce the microbial load by 5-log_10_ we found a significant exponential decrease (p = 0.004) in ALP activity.

### Fatty acids

**Figure 4:**
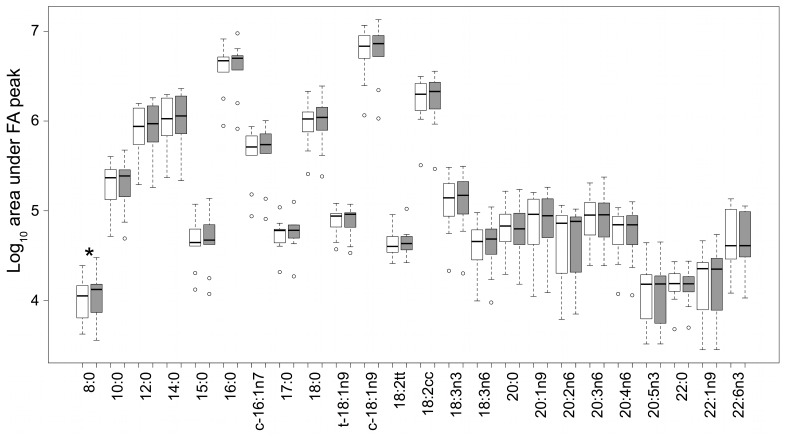
Fatty acid concentration of untreated (white) and UV-C treated with 4863 J/l (grey) samples (* p = 0.021).

Of the 24 FA tested, 19 were detected in all ten samples. The FA 17∶0, 20∶5n3, 20∶0 and 22∶1n9 were detected in nine and the FA 18∶2tt in eight samples. FA concentration had a large variation between the individual untreated human milk samples but did not significantly change during the UV-C treatment with one exception. The FA 8∶0 had a significant (p = 0.021) increase during UV-C irradiation with an untreated median area under the peak of 11,270 (range 4233 to 24,513) and 13,304 (3612 to 30,132) when treated with 4863 J/l. The FA 8∶0 was also the FA with the smallest concentration. The FA c-18∶1n9 had the largest concentration with a median area under the peak untreated of 6,819,353 (1,157,398 to 11,613,614) and treated 7,285,794 (1,067,547 to 13,459,439) (Figure 4).

## Discussion

This study is the first to show that UV-C irradiation can reduce the bacterial load in human milk to the standards required by human milk banks. Further this method retains the bioactivity of BSSL and ALP without alteration of the fatty acid profile of the human milk thus retaining factors essential to the health of both preterm and term infants.

To overcome the limited penetration of UV-C rays in opaque liquids, the micro-organisms have to be transported to the photons with the application of a turbulent flow [25]–[27]. This method has been tested on bovine milk and various fruit juices however no studies tested the method with human milk. We modified the concept used in the food industry to enable the processing of smaller volumes required for human milk. A vortical flow was used to obtain bacterial exposure to the photons in batch volumes of 380 ml. A UV-C lamp was inserted into a glass beaker containing human milk and the low velocity vortex was created to avoid damaging the human milk (Figure 1). High velocity vortex damage ranges from creating a butter-oil layer on the milk surface to the formation of butter from coalescence of milk fat globules. Therefore, it was important to select a rotation velocity that was sufficient to provide photon exposure to the micro-organisms without compromising the milk composition. Decreased bacterial viability in human milk was measured when the human milk was smoothly stirred at 500 rpm around a UV-C lamp (Figure 2).

The UV-C dosage required to reduce microbial load depended on the total solids concentration of human milk, thus dosage increased linearly with increased total solids (p<0.001) (Figure 3). However, the number of samples was too small to determine if an exponential or sigmoid curve would be a better fit than the linear. Additionally, the composition of the total solids might change the curve due to the different absorption/reflection characteristics of the different components such as fat, protein and lactose. Practically, the correct dosage to be applied to human milk can be selected in one of two ways. One, to always assume a high total solids concentration and treat with a high dosage to ensure all the bacteria are exposed to the UV-C rays. Or alternatively, the total solids concentration is measured before the treatment and dosage is adjusted according to the level of total solids concentration.

*E. coli, S. epidermidis, E. cloacae, B. cereus* and *S. aureus* reacted similarly to exposure to UV-C irradiation and required similar dosages to obtain a 5-log_10_ reduction. Our findings agree with Chang's [37] in that most vegetative bacteria have a similar resistance to UV light. A 5-log_10_ reduction in human milk is possible with this UV-C irradiation method for the five vegetative bacteria used in this study. Additionally, if the total solids concentration is known, the bacterial reduction can be controlled by adjusting the treatment time due to the high correlation of bacterial reduction and applied UV-C dosage (Figure 2).

We hypothesized that the enzymes should be minimally affected since the maximal absorbance of proteins is above the wavelength of 254 nm used for this experiment. We found no significant decrease in activity of BSSL and ALP up to a dosage of 4863 J/l required to reduce vegetative bacteria by 5-log_10_ in human milk with a very high total solid concentration. However, with a prolonged treatment time of over 23 hours (239,684 J/l), ALP was significantly reduced to 40% activity. Therefore, the decrease of ALP activity up to 4683 J/l was to small to detect with the enzymatic assay that had an intra-assay variation of 6%. These findings therefore suggest that the UV-C dosage required to reduce targeted micro-organisms in human milk does not reduce the activity of heat sensitive enzymes.

With the exception of FA 8∶0, which had the smallest concentration of all fatty acids assayed, the UV-C dosages applied in this study did not significantly alter the fatty acid profile of human milk, in terms of the total amount, the individual amounts, and the distribution of the fatty acids (Figure 4). This finding is supported by a study on goat milk were the FA profile was not significantly altered by UV-C irradiation (thin film technology) apart from the conjugated linoleic acids [38]. While it is possible that the observed change in FA 8∶0 is as a direct result of the UV-C irradiation, it may alternatively be a false positive result given that the sensitivity of the assay is poor for small fatty acid concentrations. Furthermore, the reason for the large variation in fatty acid composition within the samples (and the absence of some fatty acids in some samples) can be explained by the fact that the composition of these components are largely affected by the mothers diet [15], [16]. For example, a mother with a low-fat high-carbohydrate diet produces milk with a higher concentration of medium chain fatty acids, the milk of a mother who consumes a vegan diet contains very low concentration of DHA, while the milk of a mother who consumes a diet high in fish contains a high concentration of DHA [17].

While UV-C irradiation of drinking water is widely in use globally, the treatment of other liquids such as fruit juices is recent and no research has been carried out on human milk. This is the first study to demonstrate the potential of UV-C irradiation as a method for the pasteurization of donor human milk. The advantage that UV-C irradiation has compared to heat pasteurization methods is the retention of BSSL activity. The supply of BSSL through human milk is particularly important in preterm infants due to their low endogenous lipase activity [39]. Studies have shown that preterm infants fed pasteurized human milk have a lower fat absorption and growth rate compared to preterm infants fed raw milk [11], [12]. It is likely that the inactivation of BSSL through the thermal pasteurization process is responsible for the lower fat absorption and therefore lower growth rates in preterm infants.

### Conclusion

Experimentally, UV-C irradiation not only facilitates the required reduction in vegetative bacteria contamination to a safe level but also preserves the activity of heat sensitive enzymes such as BSSL and ALP with no change of FA thereby improving the quality of donor human milk. Nevertheless, further studies are required to investigate the effect of UV-C irradiation on other proteins such as sIgA, lactoferrin and lysozyme and on other labile components such as vitamins.
